# Adversity in early life and pregnancy are immunologically distinct from total life adversity: macrophage-associated phenotypes in women exposed to interpersonal violence

**DOI:** 10.1038/s41398-021-01498-1

**Published:** 2021-07-20

**Authors:** Kirstin Aschbacher, Melissa Hagan, Iris M. Steine, Luisa Rivera, Steve Cole, Alyssa Baccarella, Elissa S. Epel, Alicia Lieberman, Nicole R. Bush

**Affiliations:** 1 Department of Psychiatry, Weill Institute for Neurosciences, University of California, San Francisco, USA; 2 Division of Cardiology, Department of Medicine, University of California San Francisco, San Francisco, USA; 3 The Institute for Integrative Health, San Francisco, USA; 4 Department of Psychology, College of Science & Engineering, San Francisco State University, San Francisco, USA; 5 Department of Psychology, University of California, Berkeley, USA; 6 Department of Psychosocial Science, University of Bergen, Bergen, Norway; 7 Department of Anthropology, Emory University, Atlanta, Georgia; 8 Department of Psychiatry and Biobehavioral Sciences, University of California, Los Angeles, USA; 9 Department of Pediatrics, Columbia University, New York, USA; 10 Center for Health and Community, University of California, San Francisco, USA; 11 Department of Pediatrics, Division of Developmental Medicine, University of California, San Francisco, USA

**Keywords:** Predictive markers, Human behaviour, Physiology

## Abstract

Early childhood and pregnancy are two sensitive periods of heightened immune plasticity, when exposure to adversity may disproportionately increase health risks. However, we need deeper phenotyping to disentangle the impact of adversity during sensitive periods from that across the total lifespan. This study examined whether retrospective reports of adversity during childhood or pregnancy were associated with inflammatory imbalance, in an ethnically diverse cohort of 53 low-income women seeking family-based trauma treatment following exposure to interpersonal violence. Structured interviews assessed early life adversity (trauma exposure ≤ age 5), pregnancy adversity, and total lifetime adversity. Blood serum was assayed for pro-inflammatory (TNF-a, IL-1ß, IL-6, and CRP) and anti-inflammatory (IL-1RA, IL-4, and IL-10) cytokines. CD14+ monocytes were isolated in a subsample (*n* = 42) and gene expression assayed by RNA sequencing (Illumina HiSeq 4000; TruSeq cDNA library). The primary outcome was a macrophage-associated M1/M2 gene expression phenotype. To evaluate sensitivity and specificity, we contrasted M1/M2 gene expression with a second, clinically-validated macrophage-associated immunosuppressive phenotype (endotoxin tolerance) and with pro-inflammatory and anti-inflammatory cytokine levels. Adjusting for demographics, socioeconomic status, and psychopathology, higher adversity in early life (*ß* = .337, *p* = 0.029) and pregnancy (*ß* = .332, *p* = 0.032) were each associated with higher M1/M2 gene expression, whereas higher lifetime adversity (*ß* = −.341, *p* = 0.031) was associated with lower immunosuppressive gene expression. Adversity during sensitive periods was uniquely associated with M1/M2 imbalance, among low-income women with interpersonal violence exposure. Given that M1/M2 imbalance is found in sepsis, severe COVID-19 and myriad chronic diseases, these findings implicate novel immune mechanisms underlying the impact of adversity on health.

## Introduction

Exposure to adversity during sensitive periods of immunologic development is associated with heightened risk for poor health outcomes, including cardiovascular [1], metabolic [2], psychiatric [1, 3], and neurodegenerative disorders [4], as well as premature all-cause mortality [5]. Sensitive periods are limited windows of development, during which the central nervous system (CNS) and immune system can both be especially “plastic” or prone to epigenetic changes in response to environmental stimuli. “Biological embedding” [6, 7] of stress through epigenetic changes can lead to heightened health risks later in life [6–8]. While the plasticity of brain, behavior, and immunity is pronounced early in life, it continues across the adult lifespan. We know comparatively little about whether there are specific developmental stages in adulthood that are associated with heightened susceptibility to adversity and sustained phenotypic changes. Pregnancy may be an important “developmental infection point,” during which the maternal immune system undergoes profound and progressive transformations, central to the survival of mother and baby [9]. As not all women exposed to adversity in their lifetime develop poor health, a deeper understanding of the phenotypes and mechanisms associated with adversity exposure, with consideration of potential sensitive periods, is needed. Such evidence would allow development of new diagnostic biomarkers, identification of high-risk individuals, and better disease targeting [10].

Inflammation is one major pathway by which adversity exposure increases later disease risk [11]. The immune system has particular sensitive periods of development, such as early life [12, 13] and pregnancy [14–16], during which it undergoes substantial changes, exhibiting heightened phenotypic plasticity and vulnerability to environmental stressors [17–19]. Although trauma exposure tends to be associated with elevated pro-inflammatory proteins [7, 20], these biomarkers are not highly specific—i.e., they are also elevated in myriad mental and physical conditions [21–23]. Furthermore, because inflammation is multidimensional, employing a single biomarker, like C-Reactive Protein (CRP), tends not to be *sensitive* enough to optimally identify at-risk individuals [24, 25]. Accordingly, the National Institute of Health recently called for complex, composite biomarkers [26]. Inflammatory cytokines are predominantly produced by myeloid cells of the innate immune system, such as monocyte/macrophages. Hence, this study investigates whether pro-inflammatory monocyte/macrophage phenotypes may elucidate the unique biological fingerprint of adversity experienced during sensitive periods of immunological development.

Early childhood, especially the first 5 years of life, is a sensitive period during which environmental exposures influence the allocation of resources between innate (non-specific) immunity versus adaptive (specific) immunity. Exposure to adversity, especially interpersonal trauma [27], activates the neuroimmune networks that anticipate the threat of wounding and infection [28], thereby mobilizing the pro-inflammatory defenses of the innate immune system. In contrast, adaptive immunity, or targeting of specific pathogens, is more precise, but it takes years to develop [29]. It is increasingly recognized that the innate immune system can form epigenetically mediated immunologic “memory” known as “trained immunity”, which results in heightened reactions to secondary infections or sterile inflammatory triggers (like stress), and which may be intergenerationally transmissible [30–33]. Current available evidence suggests that exposure to adversity during early life drives epigenetic modifications that enhance inflammation, skew T-cell repertoires [34], and may contribute to immunosupression [8, 35, 36]. Hence, these enduring phenotypic alterations facilitate a robust inflammatory response to threats, but over time, can drive chronic disease and accelerate aging of the adaptive immune system.

As in early childhood, pregnancy is a period of an individual’s lifespan characterized by high levels of tissue proliferation, establishment of immune tolerance, and heightened vulnerability to infection—a major cause of maternal and fetal mortality [29, 37]. In apparent contradiction, pregnancy is characterized by both chronic low-grade maternal inflammation and immunosuppression, which helps restrain the inflammatory response [9, 15, 38]. Circulating blood monocytes infiltrate the uterine lining during early pregnancy, becoming “decidual macrophages”. Excess inflammation at the maternal-fetal interface is associated with a pro-inflammatory “M1-like” macrophage phenotype and predicts fetal loss and premature birth [39, 40]. In contrast, macrophages exhibiting greater expression of “M2-like” genes (immunoregulatory and anti-inflammatory) help generate immunosuppressive T-regulatory cells, which prevent rejection of the semi-allogenic (non-self) fetus [14, 15, 41]. These M1-like and M2-like profiles reflect coordinated programs involving hundreds of genes, instantiated in part through epigenetic regulation [42], whereby a relative imbalance favoring M1 versus M2-like expressed genes has been linked with inflammatory disease [43].

It may be “through the eyes” of the macrophage that pregnancy emerges as a sensitive period for immune system development in adulthood, such that the effects of adversity during pregnancy may persist post-natally. Decidual (uterine) macrophages play a pivotal role in a successful pregnancy [44]. In pregnancy, the uterus undergoes an enormous transformation “to a degree unparalleled in most adult tissues” [44]. Whereas many of her tissue macrophage compartments (e.g., the CNS) were populated when the mother herself was in utero, during her pregnancy, the decidual macrophages are largely drawn from bone marrow-derived monocytes. Under chronic stress conditions, these monocytes become biased toward M1-like, pro-inflammatory phenotypes [45–48]. Moreover, decidual macrophage phenotypes are epigenetically modified by the microenvironment [44, 49] of the fetal-maternal interface, and may be skewed toward an M1-like phenotype by stress-associated glucocorticoids [50]. In fact, psychological stress during pregnancy has been associated with leukocyte *epigenetic markers* of higher inflammation and lower T-regulatory-associated immunosuppression, which *persist after birth to predict post-partum depression* in a sample of Latinx mothers [51]. Psychosocial stress during pregnancy has also been linked to elevated expression of inflammatory genes (RNA) during pregnancy [52], which can in turn affect fetal tissue development [53] and birth timing and weight [54]. Hence, pregnancy appears to be an epigenetically sensitive window in the life of an adult woman, during which adversity may leave an enduring impact on her immune system and its reactivity to threat, as well as impacting offspring development and outcomes.

The current cross-sectional study sought to understand social adversity predictors of immune phenotypes among ethnically diverse low-income women, with significant exposure to diverse forms of interpersonal trauma. We focus on monocyte/macrophage phenotypes because they (1) exhibit developmental sensitivity during the first several years of life [13], and during pregnancy [9, 14, 15, 38], (2) drive inflammatory disease [33, 43], and (3) help regulate the balance of innate and adaptive immunity, with implications for immunosuppression [41]. Our primary hypothesis was that greater adversity during two sensitive periods of the female lifespan—early life (defined herein as birth to age five) and pregnancy—would be significantly positively associated with alterations in a macrophage-associated M1/M2 RNA phenotype, reflecting a relative upregulation of pro-inflammatory versus anti-inflammatory and immunoregulatory genes.

Furthermore, we reasoned that a novel phenotypic marker of sensitive periods should meet certain criteria for specificity with respect to the timing of exposure and the genomic fingerprint. First, we reasoned that if the M1/M2 phenotype is specific to early life or pregnancy exposures, then it should *not* be significantly associated with total adversity across the lifespan. Second, if sensitive periods are *uniquel*y associated with M1/M2, then they should not be associated with alternative biomarkers. Thus, to ascertain whether standard protein biomarkers might provide similar insights, we additionally analyzed levels of classic pro-inflammatory and anti-inflammatory cytokines and C-Reactive Protein (CRP). Similarly, we contrasted M1/M2 with an alternative macrophage-associated phenotype, known as endotoxin tolerance (ET), validated across a wide age-range among patients with sepsis [55]. We selected ET based on emerging epigenetic research that describes trained immunity and tolerance as “two opposite functional programs of innate immunity” [30], because, after an initial priming (e.g., by pathogen exposure), the former *enhances* inflammatory reactivity while the latter *suppresses* it [42].

## Methods

### Study population

Data used in the present study came from “The Child Parent Psychotherapy Health study” (CPP-HEALTH), conducted between 2013 and 2015. CPP-HEALTH enrolled 62 women and their children who were seeking treatment for the child’s exposure to interpersonal trauma. The goal was to examine biological correlates of trauma and treatment-related changes in psychological functioning following participation in a previously-validated dyadic intervention, Child-Parent Psychotherapy [56]. Inclusion criteria included biological mothers with children aged 2–6 years who had been exposed to a traumatic event, and were fluent in English or Spanish. Exclusion criteria included homelessness, current family violence, substance abuse, child was a ward of the state, current pregnancy, child developmental disorder, psychosis, and chronic medical conditions in mother or child. Maternal participants provided written consent and were compensated. The research was approved by the Institutional Review Boards at Zuckerberg San Francisco General and the University of California San Francisco.

### Participant characteristics

The present study included 53 mother–child dyads who completed the baseline assessment and provided a blood sample for inflammatory cytokine assays (Table 1). Of these, gene expression phenotypes were obtained in a convenience subset of 42. Power analyses demonstrated 80% power to detect a moderate effect size (*d* = 0.40) at *n* = 44.

**Table 1:** Sociodemographic, adversity exposure, mental health, and medical characteristics of the sample.

Sample characteristics	Serum cytokine cohort	Gene expression subcohort
	Statistical estimate (*n* = 53)	Statistical estimate (*n* = 42)
Mother’s age, years^a^	32.10 (0.92)	31.84 (0.91)
Non-Hispanic Caucasian^b^	7 (13%)	4 (10%)
Hispanic/Latina Caucasian^b^	38 (72%)	32 (76%)
African American^b^	2 (4%)	2 (5%)
Asian American^b^	6 (11%)	4 (10%)
High school education^b^	34 (64%)	24 (57%)
Family poverty^b^	35 (66%)	29 (69%)
US Born^b^	45 (85%)	37 (86%)
Child’s age, months^a^	50.38 (1.74)	49.53 (1.93)
Early life adversity^a^	0.81 (0.15)	0.81 (0.17)
Adversity in pregnancy^a^	0.91 (0.11)	0.88 (0.12)
Total life adversity^a^	11.12 (0.58)	11.58 (0.64)
PTSD severity (PSSI)^a^	21.30 (1.55)	21.81 (1.71)
Depressive symptoms (CESD-R)^a^	24.98 (1.72)	24.60 (1.82)
Body mass index^a^	27.57 (0.84)	27.53 (0.96)
Current antidepressant use^b^	6 (11%)	4 (10%)
NSAID use^b^	1 (2%)	1 (2%)

*n* = 53 constitutes the sample with serum cytokine data and *n* = 42 constitutes the subsample of these 53 women, who had gene expression data. High school education was coded 0 for participants who attended school for less than 12 years, and 1 for 12 or more years of attendance. Poverty was calculated using 2016 Census criteria (see “Methods” section).

*PSSI* PTSD Symptom Scale Interview, total score, *CESD-R* Center for Epidemiologic Studies Depression Scale, total score.

^a^Mean (SEM).

^b^*n*(%).

### Study procedures

At baseline, women provided information on their history of adversity and current psychological functioning. Participants were excluded from study enrollment if they were *currently* experiencing domestic violence. To ascertain early life adversity (ELA) and total life adversity (TLA) exposure, women completed the 30-item Life Stressor Checklist-Revised (LSC-R) in interview format [57]. Because the current study focused on interpersonal adversity, natural disasters and vehicle accidents were excluded. TLA was computed by summing the number of different types of events experienced throughout the lifespan, and ELA was quantified as the number of different types of events experienced up to age 5. Pregnancy adversity (PA) was assessed using a validated structured interview [58], in which a trained clinician asked mothers to report whether ten different types of violence (e.g., being hit in the head or stomach, choked, pushed, kicked, knifed, threatened) occurred during her pregnancy with the target child (aged 2–6 years). PA was computed as (0) no events, (1) one event, or (2) more than one event. Women also completed the Center for Epidemiologic Studies Depression Scale Revised (CESD-R) [59] and the Posttraumatic Stress Scale Interview (PSSI) [60].

### Laboratory procedures

#### Blood draw procedure

Women provided morning fasting blood samples at the hospital laboratory; prior to the draw, mothers were asked to consume nothing other than water or coffee and to reschedule if they felt ill, or had used medications in the prior three days that might affect assays/biomarkers. Serum was stored at −80°.

#### Immunogenomic phenotypes (M1/M2 ratio)

We selected a set of a priori-determined [61], previously published (44) genes reflecting an M1 and M2-like profile, quantified at baseline, based on previous literature (Supplementary Table 1). This M1/M2 phenotype reflects a relative increase of pro-inflammatory M1-like versus anti-inflammatory and immunoregulatory M2-like expressed genes. Our prior work demonstrated that higher levels of the M1/M2 phenotype prospectively predicted poorer response to behavioral trauma treatment in this same sample [62], indexed as a lesser reduction in depressive and PTSD symptoms. In line with this previous work, we computed an aggregate sum for M1-like genes and another for M2-like genes (focused on M2ab) [61], and then computed a final M1/M2 ratio as an index of relative pro-inflammatory imbalance.

#### Endotoxin tolerance (ET): an immunosuppresive phenotype

To establish whether associations of adversity during sensitive periods would be *specific to M1/M2*, or whether there might be a more general pattern of associations with other established inflammatory phenotypes, we included a previously validated, endotoxin tolerance (ET) phenotype for model comparison [55] (Supplementary Table 2). ET was specifically chosen because the profile of macrophage gene expression evoked by social stress overlaps substantially with the macrophage gene expression profile evoked by endotoxins such as lipopolysaccharides (LPS), and ET is specifically elicited in culture models by repeated LPS stimulation, hence, we hypothesized that it might provide insights into total lifetime experiences of social stress or adversity [28]. Repeated in vitro endotoxin stimulation over a 24-h period results in a temporary refractory period, during which cells exhibit sub-normal production of both pro-inflammatory and anti-inflammatory cytokines, described as “immunoparalysis.” In the context of wounding or infection, this immunoparalytic state can result in death [55]. Hence, we reasoned that greater total adversity across the lifespan might be associated with a desensitization of this negative feedback mechanism, resulting in lower ET scores.

#### Inflammatory protein profiles

The inflammatory cytokines IL-6, IL-1ß, IL-1 receptor antagonist (IL-1RA), IL-4, IL-10, and tumor necrosis factor alpha (TNF-a) were measured in serum in duplicate using a chemiluminescent multiplex assay from Meso Scale Discovery (Rockville, MD). CRP was measured using a high-sensitivity immunoturbidimetric assay from Randox (Kearneysville WV) and a PolyChem clinical chemistry analyzer (PolymedCo, Cortlandt Manor, NY). A pro-inflammatory aggregate score was comprised of the average of the normalized scores for CRP, IL-6, TNF-a, and IL-1ß. An anti-inflammatory aggregate score was constructed by averaging the normalized scores for IL-1RA, IL-4, and IL-10.

#### Immune RNA phenotyping

These methods are described elsewhere in detail (Supplementary Methods). CD14+ monocytes were isolated from total peripheral blood mononuclear cells using positive selection (anti-CD14 PE by BD Pharmingen), and frozen in RNAprotect Cell Reagent at −80°. Total RNA was isolated, converted to cDNA using with the Illumina TruSeq Stranded enzyme system and sequenced on Illumina HiSeq 4000, reads are aligned to the reference human transcriptome (HISAT2), and gene expression modeled (StringTie [63]).

### Statistical analysis

All variables were inspected for normality violations; inflammatory cytokines were blom-transformed and gene counts were log2-normalized. Multivariable regression models inputted each adversity factor as a predictor of the M1/M2 phenotype with and without adjustment for two separate sets of covariates. Model 1 included demographic and medical covariates strongly connected with inflammatory cytokines in the literature [62], and those highly characteristic of this sample: age, BMI, antidepressant use, and Hispanic ethnicity. Four participants indicated they were breastfeeding, however the small number precluded us from including it as a covariate. Model 2 included socioeconomic status factors: family poverty per census definition, being US born, and education [64]. Specificity tests for IP, pro-inflammatory (PRO) and anti-inflammatory (ANTI) cytokines were conducted similarly. Finally, we conducted multivariable regression analyses to better understand whether different adversity factors exhibited associations with immunophenotypes that were independent of one another and of psychiatric symptoms.

## Results

### Participant characteristics

On average (Table 1), women were 32 years of age (range: 22–51 years), predominantly non-white Hispanic (72%), and living in poverty (66%). The mean number of years since the adversity reported in the pregnancy recorded herein was 4 years (range: 2–6 years). Participants had been exposed to, on average, 11 different types of adverse events across their lifespan (total life adversity; TLA), with 45% reporting early life adversity (ELA), and 66% reported pregnancy adversity (PA), demonstrating high but variable levels of trauma exposure across different developmental periods. ELA and PA were not significantly correlated with one another. On average, participants exhibited elevated depressive and PTSD symptoms, were overweight, and exhibited low-grade chronic inflammation at baseline, with a mean CRP level of 2.50 (SEM: 0.42, range: 0.29–11.60) mg/L.

### Associations of adversity with immune biomarkers

**Fig. 1:**
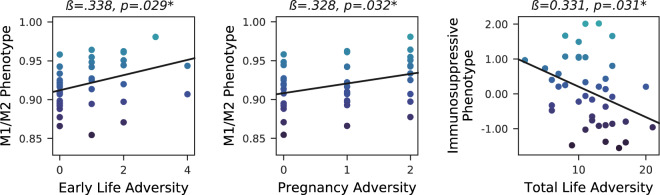
Life adversity is associated with differing immune phenotypes, depending on the timing of the exposure. Note: *p < 0.05. The scatterplots above depict the unadjusted associations between self-reported interpersonal adversity across various periods of life in relation to normalized immune gene expression signatures, with color mapped to the *y*-axis biomarker. The M1/M2 phenotype refers to a relative increase in the expression of macrophage-associated pro-inflammatory M1-like genes versus anti-inflammatory and counter-regulatory M2-like genes. The Immunosuppressive phenotype represents a tolerant or desensitized state elicited by repeated cellular stressors (e.g., endotoxin), during which time the typical inflammatory response is deactivated; lower scores imply increased duration of the inflammatory response.

ELA and PA were each significantly associated with a higher M1/M2 RNA score (all *p*’s < 0.05), whereas TLA was not (Fig. 1). Conversely, higher TLA only (but not ELA or PA) was significantly related to a lower score on the endotoxin intolerance (ET) immunosuppressive phenotype.

In contrast, neither ELA nor PA were significantly associated with serum pro-inflammatory cytokines (PRO) in any analysis (Table 2). Moreover, TLA was only significantly associated with PRO when adjusting for socioeconomic factors, such as poverty (which also exhibited a significant independent positive association with TLA), being US born, and education (both *ns*). Higher PA (but not ELA or TLA) was significantly associated with lower anti-inflammatory cytokines (ANTI) in unadjusted analyses; however, it became non-significant when adjusting for age, BMI, antidepressants, and ethnicity.

**Table 2:** Association of adversity during sensitive periods with inflammatory biomarkers.

Timing of adversity exposure	M1/M2-like phenotype			Endotoxin tolerance phenotype			Pro-inflammatory cytokines			Anti-inflammatory cytokines		
	*β*	*t*-test (df)	*p*	*β*	*t*-test (df)	*p*	*β*	*t*-test (df)	*p*	*β*	*t*-test (df)	*p*
Early life												
Unadjusted	0.337	2.265 (40)	0.029*	0.066	0.420 (40)	0.676	0.126	0.909 (51)	0.368	0.045	0.321 (51)	0.750
Adjusted model 1	0.308	2.094 (36)	0.043*	0.064	0.384 (36)	0.703	0.137	1.121 (47)	0.268	0.040	0.311 (47)	0.757
Adjusted model 2	0.311	2.114 (37)	0.042*	0.084	0.511 (37)	0.612	0.184	1.486 (48)	0.144	0.062	0.442 (48)	0.660
Pregnancy												
Unadjusted	0.332	2.226 (40)	0.032*	0.150	0.962 (40)	0.342	−0.185	−1.344 (51)	0.185	−0.333	−2.519 (51)	0.015*
Adjusted model 1	0.404	2.764 (36)	0.009**	0.180	1.061 (36)	0.296	−0.069	−0.529 (47)	0.599	−0.230	−1.745 (47)	0.087†
Adjusted model 2	0.324	2.049 (37)	0.048*	0.203	1.187 (37)	0.243	−0.123	−0.933 (48)	0.355	−0.294	−2.081 (48)	0.043*
Total lifespan												
Unadjusted	0.162	1.012 (38)	0.318	−0.341	−2.239 (38)	0.031*	0.188	1.323 (48)	0.192	0.045	0.311 (48)	0.757
Adjusted Model 1	0.054	0.315 (34)	0.755	−0.410	−2.417 (34)	0.021*	0.135	1.086 (44)	0.283	0.001	−0.001 (44)	0.999
Adjusted Model 2	0.051	0.301 (35)	0.765	−0.415	−2.544 (35)	0.016*	0.314	2.398 (45)	0.021*	0.101	0.661 (45)	0.512

**p ≤ 0.01, *p ≤ 0.05, †*p* ≤ 0.10. Separate linear regression models were fitted, each specifying one inflammatory marker as the outcome and the specified life adversity factor as the independent variable. The anti-inflammatory and pro-inflammatory aggregates include the average of the blom-standardized scores for IL-1RA, IL-4, IL-10, and IL-1B, IL-6, TNF-a, and CRP respectively. Phenotype definitions are given in the methods. Adjusted model 1 included: age, BMI, and Hispanic ethnicity and current antidepressant use as covariates; adjusted model 2 included: high school education, family poverty, and being US born. Three individuals were missing data for total life adversity.

In multivariate analyses (Table 3), ELA remained significantly associated with the M1/M2 RNA score, independent of PA and PTSD or depressive symptoms (all *p’s* < 0.05). Of note, the association of PA with M1/M2 showed a non-significant trend in the presence of ELA (*p* = 0.052). Similarly, the TLA-ET association remained significant when adjusting for potentially confounding effects of ELA and PA (*p* = 0.031), or symptoms of PTSD or depression (*p* = 0.026; Table 4). Exploratory post-hoc analyses for hypothesis generation revealed that, of the genes comprising the M1/M2 phenotype, toll-like receptor 2 (TLR2) showed the highest Pearson correlation with *both* ELA and PA (*r’s* > 0.20; Supplementary Fig. 1). Due to their exploratory nature, no correction for multiple comparisons was made.

**Table 3:** Univariate and multivariate associations of adversity during sensitive periods and mental health symptoms with the M1/M2 phenotype.

	M1/M2 phenotype		
	*β*	*t*-test	*p*-value
Univariate associations (Mental health)			
PTSD symptom severity (PSSI), df = 40	0.180	1.157	0.254
Depressive symptoms (CESD-R), df = 40	0.074	0.470	0.641
Multivariate model 1 (Adversity only, df = 39)			
Early life adversity	0.297	2.049	0.047*
Adversity in pregnancy	0.291	2.008	0.052†
Multivariate model 2 (Adversity and mental health, both)			
Early life adversity	0.330	2.171	0.036*
Adversity in pregnancy	0.312	1.916	0.063†
PTSD symptom severity (PSSI)	0.123	0.554	0.583
Depressive symptoms (CESD-R)	−0.224	−1.042	0.304

Univariate associations show that neither mental health factor is significantly associated with the phenotype in regression analyses (equivalent here to Pearson correlations). Multivariate models 1 and 2 demonstrate the relative independent associations of each sensitive period from one another and also from mental health symptoms, taking into account the small sample size may limit statistical power. Correlation of PSSI and CESD-R: *r* = 0.731, *p* < 0.001; Adversity in Early Life and Pregnancy are not significantly correlated with one another. The results of Multivariate Model 2 do not substantially change when only including either PSSI or CESD-R rather than both.

*PSSI* PTSD symptom scale interview, total score, *CESD-R* Center for epidemiologic studies depression scale, total score.

*p ≤ 0.05, †*p* ≤ 0.10.

**Table 4:** Univariate and multivariate associations of total life adversity, adversity during sensitive periods, and mental health with the endotoxin tolerance phenotype.

	Endotoxin tolerance immunosuppressive phenotype		
	*β*	*t*-test	*p*-value
Univariate associations (Mental health)			
PTSD symptom severity (PSSI), df = 40	0.115	0.730	0.470
Depressive symptoms (CESD-R), df = 40	0.189	1.216	0.231
Multivariate model 1 (df = 36)			
Total life adversity	−0.442	−2.607	0.031*
Early life adversity	0.118	0.741	0.464
Adversity in pregnancy	0.213	1.375	0.178
Multivariate model 2 (df = 36)			
Total life adversity	−0.374	−2.321	0.026*
PTSD symptom severity (PSSI)	0.034	0.143	0.887
Depressive symptoms (CESD-R)	1.182	0.805	0.426

Univariate associations show that neither mental health factor is significantly associated with the phenotype in regression analyses (equivalent here to Pearson correlations). Multivariate models 1 and 2 demonstrate the relative independent association of total life adversity from adversity during sensitive periods and from mental health symptoms.

*PSSI* PTSD symptom scale interview, total score, *CESD-R* Center for Epidemiologic Studies depression scale, total score.

*p ≤ 0.05, †*p* ≤ 0.10.

## Conclusions

Exposure to psychosocial adversity during sensitive periods of child and adult development exerts a particularly potent impact on health across the lifespan [1, 65–68]; however, the mechanisms by which adversity becomes “biologically embedded” remain elusive, and not all exposed individuals are equally at-risk. For Precision Medicine care, we need better screening tools that combine an assessment of adversity, through a developmental lens, with actionable biomarker panels [25]. Inflammation is a major mechanism that links adversity, during developmental inflection points, with increased health risks across the lifespan [8, 16–18]. Nonetheless, little research has validated diagnostic immune biomarkers that are *specific* to adversity during two crucial sensitive periods of a woman’s lifespan [69]—her early life and her own pregnancy—during which her immune system undergoes significant transformation. This study leverages a unique, cross-sectional clinical sample of low-income, ethnically diverse women with a history of exposure to interpersonal violence, to identify associations between adversity during early life and pregnancy, in comparison to total lifetime exposure, with current immune phenotypes.

This study identifies the macrophage-associated M1/M2 phenotype as a specific biomarker of exposure to adversity during early life (ELA) and during pregnancy (PA). Specifically, we found that greater ELA and greater PA were associated with a greater relative expression of M1 versus M2-like genes in CD14+ circulating monocytes, indicating a pro-inflammatory imbalance, even when adjusting for sociodemographic, medical, and psychological factors. Adding confidence to the *specificity* of M1/M2 as a biomarker of adversity during these two sensitive periods, the M1/M2 phenotype was *not* significantly associated with total lifetime adversity (TLA) exposure.

Macrophages are the primary cellular mediators of inflammation, a complex response to actual and anticipatory threats [28], involving coordinated transcriptional programs across hundreds of expressed genes [70]. Hence, inflammation cannot be meaningfully understood by one or two serum protein biomarkers. During the arc of an inflammatory response, marophage phenotypes are highly dynamic, transitioning from a pro-inflammatory M1 (classically activated) phenotype across a spectrum of anti-inflammatory/immunoregulatory M2-like (alternatively activated) phenotypes [61, 70]. Prior evidence demonstrates that a pro-inflammatory M1/M2 polarization skew is associated with heightened risk for myriad adverse clinical outcomes, including atherosclerosis, diabetes, neurodegeneration, pregnancy complications, and mental health treatment non-response [14, 43, 61, 62, 71–73]. As such, these findings demonstrating potential sensitive windows of development in the etiology of a pro-inflammatory M1/M2 phenotype have significant potential for advancing understanding of the origins of myriad disease risks.

In contrast, adversity during sensitive periods did *not* significantly correlate with a pro-inflammatory aggregate score, composed of CRP, IL-6, TNF-a, and IL-1ß. Despite other studies linking early life adversity and CRP [74], in the present study, only total lifetime adversity correlated with the pro-inflammatory protein profile, and only when adjusting for socioeconomic factors. Notably, the average level of the pro-inflammatory protein CRP in this sample was 2.5 mg/L (range: 0.29–11.60), which indicates prevalent low-grade inflammation associated with heightened cardiovascular risk [75]. As such, while the small sample size may have limited statistical power, nonetheless, there was good statistical distribution in the pro-inflammatory outcome, and the effect sizes were small (*β* < 0.20), suggesting that the M1/M2 phenotype may be a more sensitive biomarker than serum proteins.

The present findings paint pregnancy as a sensitive period in a woman’s life span for bioembedding of experience within her own immune system, rather than solely as a “vessel” for the fetus. It is established that balance of maternal M1 versus M2 phenotypes of decidual macrophages in the uterine lining plays a crucial role in a successful pregnancy, from implantation, to fetal tolerance, to parturition [14]. Other studies have reported that stress during pregnancy is cross-sectionally associated with a pro-inflammatory milieu and markers of impaired adaptive immunity [17]. In this study, women who retrospectively reported greater adversity during pregnancy not only had an increased M1/M2 skew, but also had *lower* levels of anti-inflammatory cytokines, at least two, and as many as 6 years after the pregnancy ended. Furthermore, this correlation was not better explained by total life adversity, which included assessment of recent stressful events. At least one prior study has reported that adversity during pregnancy was associated with epigenetic alterations consistent with an M1/M2 imbalance that persisted post-natally [51]. A second study also linked psychosocial stress during pregnancy (but not prior to pregnancy) with elevated inflammatory gene expression profiles consistent with M1/M2 imbalance [52]. Moreover, these stress-related material immunologic alterations appear to impact fetal tissue development and immune regulation [53, 54] in ways that can potentially shape neonatal development. Altogether, these findings align with recent research highlighting women’s neurobiological plasticity during pregnancy and the potential for biological embedding of stress during this time, for the mother herself, as well as for her baby [76].

These findings revealed an intriguing contrast between two macrophage-associated phenotypes with potential implications for the discovery of novel diagnostic biomarkers, experimental paradigms, and treatment approaches [77]. First, adversity during sensitive periods (but not total life adversity) was associated with an *increased* pro-inflammatory M1/M2 phenotype skew. Second, total adversity across the life span was associated with *reduced* expression of an immunosuppressive, endotoxin tolerance phenotype, validated among adults and children with sepsis [55]. Futhermore, this association remained significant, even when adjusting for sensitive period exposure.

To provide context for the significance of these findings, the body’s defense against infection balances “host resistance” with “disease tolerance”. Resistance kills or clears pathogens (e.g., via inflammatory responses), whereas, tolerance helps limit tissue damage (e.g., via prolonged inflammation) [78], and is a response to repeated exposure [79, 80]. Experimental models using a *single* endotoxin exposure have provided significant insights into inflammation as an important pathway by which adversity leads to mental and physical health risks [81, 82]. However, to our knowledge, this is the first time a human study has reported an association of any psychosocial factor with an *endotoxin tolerance phenotype, a novel translational model for repeated adversity exposures*. These findings extend prior evidence in mice reporting that chronic unpredictable stress suppressed the development of LPS-induced endotoxin intolerance, and induced heightened pro-inflammatory markers in the hippocampus [83]. Our findings may reflect a “weathering effect*”* [84], such that a lifetime of recurrent adversity may blunt tolerance mechanisms of cellular resilience and enable more reactive or prolonged inflammatory responses, thereby potentially accelerating disease risk.

We speculate that the association between adversity exposure during sensitive periods and the M1/M2 phenotype might be an adaptive manifestation of “trained immunity” [30, 78], a long-lasting, epigenetically-mediated form of innate immunologic memory. The M1/M2 phenotype may reflect a bias toward greater M1-mediated resistance and lower M2-mediated tolerance, although future studies would ideally validate the functional phenotype. This study’s specific M2-like signature closely follows the so-called M2a and M2b subtypes [61]. Hence, this M1/M2 signature reflects not only increased inflammatory mediators, but reduced expression of genes that code for several critical functions: (1) “self-recognition” complexes and T-helper cell signaling (Human Leukocyte Antigen-DR isotype (HLA-DR), CD80 and CD86), (2) critical activators of immunosuppressive T-regulatory cells (transforming growth factor-beta (TGF-β) and IL-10), and (3) cytokines/receptors that *inhibit* IL-1β-mediated inflammation (IL-1RA, IL-1R2, and IL-10) [15, 85, 86]. Reduced expression of these M2-like genes suggests that cross-talk with adaptive immune cells may be reduced, particularly immunsuppressive T-regulatory cells and T-helper cells that orchestrate the adaptive immune repertoire [87].

Crucially, although trained immunity is typically studied in vaccination and infection models, it relies on cellular stress and damage pathways that can be activated by psychological stress [28]. Exciting new evidence suggests that high catecholamine can also induce trained immunity in human monocytes both in vitro and in vivo [88], resulting in epigenetic markers (H3K4me3), as seen in vaccination models [30]. In that study, in vivo norepinephrine induction did not explicitly utilize an in vivo experimental stress model, providing an important direction for future research. Another cardinal characteristic of trained immunity is a heightened pro-inflammatory response to challenge, after an initial priming exposure [30]. Among humans and animals in vivo, experimental psychological stress tests acutely increase secretion of M1-associated pro-inflammatory and pro-atherogenic cytokines like IL-1β [82, 89] via sympathetic arousal, and enhance the binding of nuclear factor kappa-B (NF-kB) in peripheral leukocytes [28], a transcriptional regulator that induces a pro-inflammatory M1-like phenotype. Conversely, relative decreases in M2-associated IL-10, as suggested by this M1/M2 phenotype, may reduce tolerance and delay stress recovery. Consistent with the functional phenotype expected if adversity during sensitive periods results in trained immunity and long-lasting innate immunologic memory, depressed individuals with early life adversity exhibited greater inflammatory reactivity to a standardized acute stress task than similarly depressed individuals without early adversity [90]. Hence, we posit that adversity during sensitive periods “educates” the macrophage to mount a heightened inflammatory response, whereas repeated life adversity “wears down” or desensitizes the mechanisms that *constrain* the inflammatory response. Through a developmental perspective, these findings might suggest that an M1/M2 skew associated with adversity during sensitive periods may reflect an anticipatory adaption (intelligent resource allocation), whereas reduced ET associated with total lifetime adversity may reflect a reactionary adaption (limiting damage induced by weathering).

### Strengths and limitations

These data come from a sample of diverse, low-income women with high exposure to violence and other adversities, and findings may not generalize to men or different populations of women. As a limitation, women were interviewed about adversity experienced during one particular pregnancy due to clinic-based recruitment; hence, possible additional adversity during other pregnancies was not assessed. Although other sensitive periods of development may be of interest, available research suggests monocyte/macrophages are likely to be most developmentally sensitive during early life [13] and during pregnancy [9, 14, 51]. We acknowledge that in vivo macrophage phenotypes are dynamic, multidimensional, and tissue-specific [70, 91] and may differ from over-simplified in vitro frameworks of the M1/M2 spectrum. Future studies should triangulate gene expression phenotypes with cell surface and functional phenotyping. Single cell RNA seq would be needed to resolve whether M1 and M2 expression is co-expressed on single cells or represents a shift in underlying myeloid lineage cell subsets.

We now understand that adversity exposure is as veritable a risk factor for chronic disease as poor diet or exercise; yet, we understand little about the immunological mechanisms that help explain this link. This study identifies the M1/M2 RNA phenotype as a novel and specific adulthood biomarker of adversity exposure during two sensitive developmental periods in the female lifespan and points to paths for advancing translational research targeting these mechanisms. Such discoveries may further Precision Medicine through developmentally-oriented adversity screening paired with sophisticated biomarker panels to support individuals and prevent adversity-associated morbidity.

## Supplementary information

Supplemental Text, Table, Figure

## Data Availability

FASTQ files are available from the NCBI Sequence Read Archive (SRA) under BioProject PRJNA626346.
