# Supplementary material for: Adversity in early life and pregnancy are immunologically distinct from total life adversity: macrophage-associated phenotypes in women exposed to interpersonal violence
Source: Transl Psychiatry. 2021 Jul 20;11:391. doi: 10.1038/s41398-021-01498-1 (PMC8289995; doi:10.1038/s41398-021-01498-1)
Supplement: Supplementary file 1 — Supplemental Text, Table, Figure [file 41398_2021_1498_MOESM1_ESM.docx]

**Supplementary Material**

**Supplementary Methods**

**Supplementary Table 1. Gene Expression Associated with M1 and M2-like Polarization Phenotypes**

**Supplementary Table 2. Gene Expression Associated with IP**

**Supplementary Figure 1. Toll-like Receptor 2 (TLR2) at the Nexus of Adversity During Sensitive Periods**

**Supplementary References**

**Supplementary Methods**

**CD14+ Isolation and RNA Isolation**

As previously described (REF), we collected 30mL blood for PBMC isolation into Sodium-Heparin Vacutainer Cell Preparation Tubes (CPT, BD Biosciences Cat #362753), and counted PBMCs on an Accuri C6 volumetric cytometer (BD Biosciences), with 3x10^7^ cells used for downstream purification. EasySep PE Selection Kit (Stem Cell Technologies Cat# 18551) was employed to isolate CD14+ cells by positive selection according to the manufacturer’s protocol (anti-CD14 PE by BD Pharmingen). After enrichment with 3 wash steps, CD14+ cells were resuspended in MACS buffer and between 0.5x10^6^ to 10x10^6^ CD14+ cells were centrifuged and resuspended/lysed (with 600uL of RNAprotect Cell Reagent) for RNA-seq, and quickly stored at -80°. We used flow cytometry to conduct quality control assessments of pre-purity, post-purity, and CD14+ contamination of the negative fractions.

**RNA Sequencing**

Total RNA was isolated from CD14+ cells (Qiagen RNeasy), investigated for sufficient mass (>100 ng by RiboGreen RNA) and integrity (RIN >8 by Agilent TapeStation capillary electrophoresis), and subsequently converted to cDNA (with the Illumina TruSeq Stranded enzyme system). Subsequently, cDNA libraries were sequenced on an Illumina HiSeq 4000 instrument (UCLA Neuroscience Genomics Core Laboratory), according to the manufacturers’ standard protocols. This sequencing targeted >20 million, 65-base pair, single-stranded reads, with depths averaging 33.8 million reads per sample.

**Read Alignment and Expression Modeling**

We aligned the demultiplexed reads (FASTQ format) with the reference human transcriptome (HG38) using HISAT2 ^1,2^, and modeled transcript and gene expression with StringTie^1,3^. FASTQ files are available from the NCBI Sequence Read Archive (SRA) under BioProject PRJNA626346. Gene expression values were quantified as log2-transformed transcripts per million. After quality-assurance analyses of RNA-seq data, one sample was excluded from downstream analysis given its low RIN number of 3, which was a definitive outlier on principal component analysis.

**Supplementary Table 1. Gene Expression Associated with M1 and M2-like Polarization Phenotypes**

| **Gene Symbols** |  | **Full Names** |
| --- | --- | --- |
| **M1-Associated** |  |  |
| CD80 |  | Cluster of differentiation 80 |
| CD86 |  | Cluster of differentiation 86 |
| IL1R1 |  | Interleukin 1 Receptor Type 1 |
| TLR2 |  | Toll Receptor 2 |
| TLR4 |  | Toll Receptor 4 |
| NOS2 (iNOS) |  | Inducible Nitric Oxide Synthase |
| **M2ab-Associated** |  |  |
| CD163 |  | Hemoglobin Scavenger Receptor |
| SCARB1 (SR) |  | Scavenger Receptor Class B Member 1 |
| MRC1 (CD206) |  | Mannose Receptor C-Type 1 |
| NR3C2 (MR) |  | Nuclear Receptor Subfamily 3 Group C Member 2 (Mineralcorticoid Receptor) |
| IL1R2 |  | Interleukin 1 Receptor Type 2 |
| ARG1 |  | Arginase 1 |
| IL10 |  | Interleukin 10 |
| IL1RN |  | Interleukin 1 Receptor Antagonist |
| TGFB1 |  | Transforming Growth Factor Beta 1 |
| CD86 |  | CD86 Molecule |
| CCL1 |  | C-C Motif Chemokine Ligand 1 |
| HLA-DRA |  | MHC II, DR Alpha |
| HLA-DRB1 |  | MHC II, DR Beta 1 |
| HLA-DRB5 |  | MHC II, DR Beta 5 |
| HLA-DRB6 |  | MHC II, DR Beta 6 (Pseudogene) |
| HLA-DRB9 |  | MHC II, DR Beta 9 (Pseudogene) |

Note: MHC II = Major Histocompatibility Complex, Class II. Gene selection and symbols informed by Labonte et al., (2014) and https://www.genecards.org. CD86 is listed by Labonte et al., as being associated with both an M1-like and M2b-like phenotype; hence, it occurs in both categories herein. YM1/CHIA and FIZZ1/RETNLB were not included because >90% of the observations had a count of zero.

**Supplementary Table 2. Gene Expression Associated with IP**

| **Gene Symbols** |  | **Full Names** |
| --- | --- | --- |
| CD19 |  | Coreceptor for the B-cell antigen receptor complex on B lymphocytes |
| CCL22 |  | C-C motif chemokine 22 |
| CD14 |  | Cluster of differentiation 14 |
| CD300LF |  | CMRF35-like molecule 1 |
| CYP1B1 |  | Cytochrome P450 1B1 |
| DHRS9 |  | Dehydrogenase/reductase SDR family member 9 |
| FCER1G |  | Fc fragment of IgE, high affinity I, receptor for; gamma polypeptide |
| FPR1 |  | N-formyl peptide receptor 1 |
| FPR2 |  | N-formyl peptide receptor 2 |
| GK |  | Glycerolkinase |
| HK2 |  | Hexokinase 2 |
| HK3 |  | Hexokinase 3 |
| HPSE |  | Heparanase |
| LILRA5 |  | Leukocyte Immunoglobulin Like Receptor A5 |
| MGST1 |  | Microsomal Glutathione S-Transferase 1 |
| PDLIM7 |  | LIM Mineralization Protein |
| PLAUR |  | Plasminogen Activator, Urokinase Receptor |
| PSTPIP2 |  | Proline-Serine-Threonine Phosphatase Interacting Protein 2 |
| RAB13 |  | RAB13, Member RAS Oncogene Family |
| RETN |  | Resistin |
| RHBDD2 |  | Rhomboid Domain Containing 2 |
| S100A4 |  | S100 Calcium Binding Protein A4 |
| S100A9 |  | S100 Calcium Binding Protein A9 |
| S100A12 |  | S100 Calcium Binding Protein A19 |
| SERPINA1 |  | Serpin Family A Member 1 |
| CPVL* |  | Carboxypeptidase Vitellogenic Like |
| CST3* |  | Cystatin C |
| Ly86* |  | Lymphocyte Antigen 86 |
| PROCR* |  | Protein C Receptor |

Note: * down-regulated, all other are up-regulated. HIST2H2AA3 was not included because there was no variance in this sample. CD19orf59 could not be found in the dataset.

**Supplementary Figure 1. Toll-like Receptor 2 (TLR2) at the Nexus of Adversity During Sensitive Periods**

Note: In a post-hoc analysis for hypothesis generation, we inquired how many of the individual genes out of the M1/M2 and Immunosuppressive signatures were correlated with Early Life Adversity and Pregnancy Adversity at a |threshold| >.20, which we chose to represent a small effect size. Only TLR2 met these criteria, which is concordant with prior literature highlighting a role for TLR2 in early adversity, metabolism, and potentiation of TLR4-mediated inflammation (see Conclusions). TLR2 was correlated with ELA (r=.238, p=.128) and with PA (r=.304, p=.050). The fact that these correlations are still low, and in the case of ELA, non-significant, underscores the fact that these gene expression signatures (M1/M2 and immunosuppression) represent complex, multidimensional biomarkers, the predictive value of which are driven by the combination of small individual contributions of many genes together, rather than being driven by a few very highly correlated genes.

**Supplementary References**

1. Pertea M, Kim D, Pertea GM, Leek JT, Salzberg SL. Transcript-level expression analysis of RNA-seq experiments with HISAT, StringTie and Ballgown. *Nat Protoc*. 2016;11(9):1650-1667. doi:10.1038/nprot.2016.095

2. Kim D, Langmead B, Salzberg SL. HISAT: a fast spliced aligner with low memory requirements. *Nat Methods*. 2015;12(4):357-360. doi:10.1038/nmeth.3317

3. Pertea M, Pertea GM, Antonescu CM, Chang T-C, Mendell JT, Salzberg SL. StringTie enables improved reconstruction of a transcriptome from RNA-seq reads. *Nat Biotechnol*. 2015;33(3):290-295. doi:10.1038/nbt.3122
